# 16S rRNA Methylase–producing, Gram-Negative Pathogens, Japan

**DOI:** 10.3201/eid1304.060501

**Published:** 2007-04

**Authors:** Kunikazu Yamane, Jun-ichi Wachino, Satowa Suzuki, Naohiro Shibata, Haru Kato, Keigo Shibayama, Kouji Kimura, Kumiko Kai, Satoshi Ishikawa, Yoshiyuki Ozawa, Toshifumi Konda, Yoshichika Arakawa

**Affiliations:** * National Institute of Infectious Diseases, Tokyo, Japan

**Keywords:** Aminoglycoside resistance, 16S rRNA methylase, gram-negative bacteria, dispatch

## Abstract

To investigate the exact isolation frequency of 16S rRNA methylase–producing, gram-negative pathogenic bacteria, we tested 87,626 clinical isolates from 169 hospitals. Twenty-six strains from 16 hospitals harbored 16S rRNA methylase genes, which suggests sparse but diffuse spread of pan-aminoglycoside–resistant microbes in Japan.

Broad-spectrum β-lactams and fluoroquinolones have been widely prescribed in the treatment of gram-negative bacterial infections; as a result, resistance to these antimicrobial agents has developed in some species. Although these agents are not immune to an increasing number of resistance mechanisms, they remain relatively potent and continue to be essential antimicrobial drugs for treating life-threatening bacterial infections.

Although the production of aminoglycoside-modifying enzymes is the most common mechanism of resistance in aminoglycosides, the emergence of pan-aminoglycoside–resistant, 16S rRNA methylase–producing, gram-negative bacteria has been increasingly reported in recent years. Five types of plasmid-mediated 16S rRNA methylases (ArmA, RmtA, RmtB, RmtC, and RmtD) have so far been identified in east Asia, Europe, and South America (*1*–*7*). RmtA was first identified in 2001 in Japan (*3*) and has so far been identified exclusively in *Pseudomonas aeruginosa* (*8*). RmtC was subsequently identified only in *Proteus mirabilis* (*4*). RmtB has been found among various gram-negative bacterial species, including *Serratia marcescens*, *Escherichia coli*, *Citrobacter freundii*, *Klebsiella pneumoniae*, and *K. oxytoca,* isolated in Japan, South Korea, and Taiwan (*2*,*5*,*6*,*9*). Another new 16S rRNA methylase was initially identified in *C. freundii* in Poland, submitted to European Molecular Biology Laboratory (EMBL)/GenBank in 2002 (accession no. AF550415), and later characterized and assigned as ArmA in *K. pneumoniae*, *E. coli*, *Enterobacter cloacae*, *Salmonella enterica*, and *Shigella flexneri* in France, Bulgaria, and Spain (*10*,*11*). Moreover, ArmA was also identified in *E. coli*, *K. pneumoniae*, *E. cloacae*, *C. freundii* and *S. marcescens* in South Korea, Japan, and Taiwan (*5*,*8*,*9*). This enzyme has also been identified in a glucose nonfermentative *Acinetobacter* sp. in South Korea and Japan (*6*,*8*). Quite recently, RmtD was newly identified in the SMP-1–producing *P. aeruginosa* strain PA0905, which was isolated in Brazil (*7*). In Japan, arbekacin, a semisynthetic aminoglycoside, has been approved for treatment of methicillin-resistant *Staphylococcus aureus* infections, and this agent is also very efficacious for gram-negative bacteria. However, 16S rRNA methylase–producing microbes can adapt to this agent, and its prescription may well be a selective pressure on the kind of microbes in the clinical environment. Thus, this investigation was conducted to determine the exact isolation frequency of 16S rRNA methylase–producing, gram-negative pathogenic bacteria in Japanese medical facilities and assess the possibility of the future prevalence of these hazardous microbes.

## The Study

From September 1 to October 31, 2004, 169 medical facilities with in-house microbiology laboratories participated in this investigation. Clinical specimens were collected from inpatients and outpatients with suspected infections. Bacterial isolates that belonged to the family *Enterobacteriaceae* or were nonfermentors of glucose, for example, *P*. *aeruginosa* and *Acinetobacter* spp., were included in this study. A total of 87,626 clinical isolates were collected and analyzed. The results are shown in Table 1.

**Table 1:** Gram-negative strains investigated during September and October, 2004

Bacterial species	Strains, n			Rate of 16S rRNA methylase-producing strains, %
	Isolated	Resistant to all aminoglycosides tested	Harboring 16S rRNA methylase gene, n	
Pseudomonas aeruginosa	18,037	384	14	0.08
Escherichia coli	14,701	39	3	0.02
Klebsiella spp.	12,293	11	1	0.008
Enterobacter spp.	6,398	26	2	0.03
Acinetobacter spp.	3,116	33	4	0.12
Serratia marcescens	3,009	14	0	0
Citrobacter spp.	2,422	1	0	0
Proteus spp.	2,389	8	2	0.08
Alcaligenes spp.	443	0	0	0
Other	24,818	8	0	0
Total	87,626	527	26	0.03

**Figure 1:**
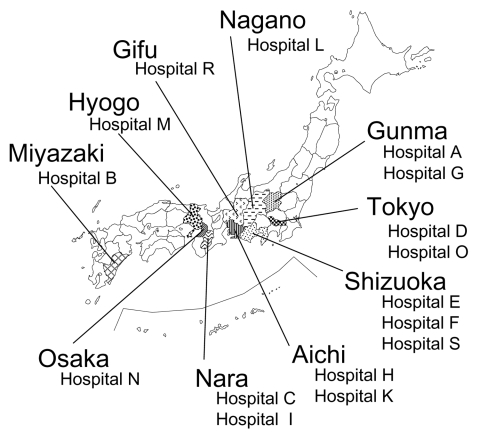
Geographic distribution of hospitals where 16S rRNA methylase gene–positive strains were isolated. Of 16 hospitals, 8 were located in the Kanto area (Gunma, Tokyo, Shizuoka, and Nagano), 3 in the Chubu area (Aichi and Gifu), 4 in the Kinki area (Osaka, Nara, and Hyogo), and 1 in the Kyushu area (Miyazaki). This distribution suggests a sparse but diffuse spread of 16S rRNA methylase–producing, gram-negative pathogenic microbes in Japan. Bacterial species and type of 16S rRNA methylase identified in each hospital are shown in Table 3.

Twenty-nine strains (17 *P*. *aeruginosa*, 4 *A*. *baumannii*, 3 *E*. *coli*, 2 *P*. *mirabilis*, 1 *E*. *cloacae*, 1 *K. pneumoniae*, and 1 *Enterobacter aerogenes*) that grew on LB agar plates supplemented with 500 mg of arbekacin per liter were subjected to the typing of 16S rRNA methylase genes by a multiplex PCR. Primers used for the PCR amplification of bacterial 16S rRNA methylase genes were the following: RMTA-F 5′-CTA GCG TCC ATC CTT TCC TC-3′ and RMTA-R 5′-TTT GCT TCC ATG CCC TTG CC-3′, which amplify a 635-bp DNA fragment within *rmtA* gene (*3*); RMTB-F 5′-GCT TTC TGC GGG CGA TGT AA-3′ and RMTB-R 5′-ATG CAA TGC CGC GCT CGT AT-3′, which amplify a 173-bp DNA fragment within *rmtB* (*2*); RMTC-F 5′-CGA AGA AGT AAC AGC CAA AG-3′ and RMTC-R 5′-ATC CCA ACA TCT CTC CCA CT-3′, which amplify a 711-bp DNA fragment within *rmtC* (*4*); and ARMA-F 5′-ATT CTG CCT ATC CTA ATT GG-3′ and ARMA-R 5′-ACC TAT ACT TTA TCG TCG TC-3′, which amplify a 315-bp DNA fragment within *armA* (accession nos. AY220558 and AB117519). PCR results and clinical data from these 29 strains are summarized in the Table 2. Genes for 16S rRNA methylases were absent in 3 arbekacin high-level-resistant strains of *P. aeruginosa* by PCR analyses that used 4 sets of 16S rRNA methylase-specific primers. In these strains, simultaneous production of multiple aminoglycoside-modifying enzymes was suggested as reported previously (*12*). Twenty-six strains harboring any of the four 16S rRNA methylase genes were identified in 16 hospitals, with no apparent geographic convergence in the locations of the hospitals (Figure 1). In hospital L, 3 different bacterial species (*E. coli*, *E. aerogenes,* and *K. pneumoniae*) harbored the *armA* gene, which suggests probable conjugal transfer of *armA*-carrying plasmids among different bacterial species.

**Table 2:** Bacterial species and type of 16S rRNA methylase gene detected*

Strain no.	Bacterial species	PCR type of 16S rRNA methylase gene	Hospital	Clinical specimen
40	Proteus mirabilis	*rmtC*	A	Sputum
64	*Pseudomonas aeruginosa*	*rmtA*	B	Sputum
101	*P. aeruginosa*	*rmtA*	C	Otorrhea
103	*P. aeruginosa*	*rmtA*	C	Otorrhea
109	*P. aeruginosa*	*rmtA*	C	Otorrhea
113	*P. aeruginosa*	*rmtA*	D	Bile
127	*P. aeruginosa*	*rmtA*	D	Pharynx
157	*P. aeruginosa*	*rmtA*	D	Pharynx
158	*P. aeruginosa*	*rmtA*	D	Stool
231	*Acinetobacter baumannii*	*armA*	E	Wound
249	*P. aeruginosa*	*rmtA*	F	Pus
252	*P. aeruginosa*	*rmtA*	F	Pleural fluid
328	*P. mirabilis*	*rmtC*	G	Sputum
353	*P. aeruginosa*	*rmtA*	H	Sputum
386	*Escherichia coli*	*rmtB*	I	Urine
422	*P. aeruginosa*	UD	J	Urine
463	*P. aeruginosa*	*rmtA*	K	Urine
469	*E. coli*	*armA*	L	Skin
470	*Enterobacter aerogenes*	*armA*	L	Stool
471	*Klebsiella pneumoniae*	*armA*	L	Stool
479	*P. aeruginosa*	*rmtA*	M	Unknown
499	*E. coli*	*armA*	N	Urine
509	*Enterobacter cloacae*	*armA*	O	Urine
525	*P. aeruginosa*	UD	P	Urine
527	*P. aeruginosa*	UD	Q	Blood
593	*P. aeruginosa*	*rmtA*	R	Vaginal secretion
615	*A. baumannii*	*armA*	S	Sputum
617	*A. baumannii*	*armA*	S	Sputum
619	*A. baumannii*	*armA*	S	Pus

*Strains for which MIC of arbekacin was 512 mg/L or greater are listed; UD, undetected.

**Figure 2:**
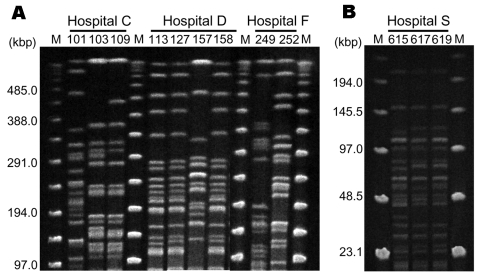
A) Pulsed-field gel electrophoresis (PFGE) fingerprinting patterns of *Spe*I-digested total DNA preparations from *Pseudomonas aeruginosa*. M, Lambda ladder PFGE molecular mass marker (Bio-Rad, Hercules, CA, USA). Strains 103 and 109 show similar patterns, which suggests probable nosocomial transmission of *rmtA*-positive strains in hospital C. Strains 113, 127, and 158 also demonstrate similar patterns, which implies possible nosocomial transmission in hospital D. However, 2 different PFGE patterns are observed in hospitals C, D, and F, which suggests transfer of plasmids carrying 16S rRNA–methylase genes among *P. aeruginosa* strains with different genetic backgrounds. B) *Sma*I-digested total DNA preparations from *Acinetobacter baumannii* isolated from hospital S. Three strains demonstrate the same PFGE pattern, which suggests probable nosocomial transmission of *armA*-positive *A. baumannii* in hospital S. M, lambda ladder low-range PFGE molecular mass marker (New England Biolabs, Ipswich, MA, USA).

Pulsed-field gel electrophoresis (PFGE) was performed on 9 strains of *P*. *aeruginosa* and 3 strains of *A. baumannii* isolated from 4 separate hospitals where 16S rRNA methylase genes were isolated (Figure 2). Genomic DNA preparations from *P*. *aeruginosa* and *A. baumannii* were digested with *Spe*I and *Sma*I, respectively. Clonality was inferred based on the criteria of Tenover et al. (*13*) Two of 3 *rmtA*-positive *P. aeruginosa* strains isolated in hospital C were estimated to be the same clone. Among 4 *rmtA*-positive *P. aeruginosa* isolates recovered in hospital D, 2 different clonal lineages were observed. This finding suggests possible conjugal transfers of *rmtA*-carrying plasmids among genetically different strains of *P. aeruginosa*. Three *armA* gene–harboring *A. baumannii* identified in hospital S were obviously the same clone. These findings imply probable nosocomial transmission of 16S rRNA methylase gene–harboring strains in hospitals C, D, and S, as well as frequent conjugal transfers of plasmids carrying 16S rRNA methylase genes among gram-negative pathogenic bacterial species.

MIC determinations were performed according to the guideline of the CLSI (formerly National Committee on Clinical Laboratory Standards). All 16S rRNA methylase-positive strains were highly resistant (MICs >1,024 mg/L) of all 4,6-disubstituted deoxystreptamine group aminoglycosides (Table 3). In contrast, resistance to streptomycin and neomycin varied. Three16S rRNA methylase gene-negative *P*. *aeruginosa* strains were also highly resistant to arbekacin, but the MICs of some of the 4,6-disubstituted deoxystreptamine group aminoglycosides were relatively lower (256–512 mg/L) for these strains than those for 16S rRNA methylase gene–positive strains (>1,024 mg/L). Strains harboring 16S rRNA methylase genes tended to show resistance to oxyimino-cephalosporins such as cefotaxime and ceftazidime as well, but were susceptible to imipenem. As reported for the *armA*- or *rmtB*-bearing strains, the presence of β-lactamase genes was suggested in cefotaxime-resistant strains, and indeed the *bla*_CTX-M-14_ gene was detected in several *rmtB*-positive strains tested in our study (data not shown). Some of these strains also demonstrated resistance to fluoroquinolones (Table 3).

**Table 3:** MICs of antimicrobial agents for arbekacin-resistant strains*†‡

Strain no.	MIC (mg/L)										
	ABK	AMK	TOB	ISP	GEN	SM	NEO	CTX	CAZ	IPM	CIP
40	>1,024	>1,024	>1,024	>1,024	>1,024	8	>1,024	<0.06	0.125	0.125	64
64	>1,024	>1,024	>1,024	>1,024	>1,024	8	>1,024	<0.06	0.5	0.125	64
101	>1,024	>1,024	>1,024	>1,024	>1,024	32	>1,024	8	2	0.5	32
103	>1,024	>1,024	>1,024	>1,024	>1,024	32	16	64	2	0.5	<0.06
109	>1,024	>1,024	>1,024	>1,024	>1,024	8	16	64	16	0.5	<0.06
113	>1,024	>1,024	>1,024	>1,024	>1,024	128	512	16	2	16	0.125
127	>1,024	>1,024	>1,024	>1,024	>1,024	128	128	16	2	16	<0.06
157	>1,024	>1,024	>1,024	>1,024	>1,024	32	32	64	4	2	0.5
158	>1,024	>1,024	>1,024	>1,024	>1,024	128	512	32	8	16	0.125
231	>1,024	>1,024	>1,024	>1,024	>1,024	>1,024	32	>128	128	4	16
249	>1,024	>1,024	>1,024	>1,024	>1,024	256	512	16	1	4	<0.06
252	>1,024	>1,024	>1,024	>1,024	>1,024	512	512	128	4	4	8
328	>1,024	>1,024	>1,024	>1,024	>1,024	8	512	>128	>128	2	32
353	>1,024	>1,024	>1,024	>1,024	>1,024	32	256	64	>128	4	32
386	>1,024	>1,024	>1,024	>1,024	>1,024	256	256	128	>128	0.5	>128
422	>1,024	>1,024	>1,024	256	>1,024	512	>1,024	>128	>128	8	128
463	>1,024	>1,024	>1,024	>1,024	>1,024	64	128	16	4	8	32
469	>1,024	>1,024	>1,024	>1,024	>1,024	64	32	>128	8	0.25	<0.06
470	>1,024	>1,024	>1,024	>1,024	>1,024	128	8	>128	>128	4	1
471	>1,024	>1,024	>1,024	>1,024	>1,024	64	8	128	4	0.25	<0.06
479	>1,024	>1,024	>1,024	>1,024	>1,024	256	1,024	64	4	0.25	0.25
499	>1,024	>1,024	>1,024	>1,024	>1,024	64	4	0.06	0.125	0.25	0.25
509	>1,024	>1,024	>1,024	>1,024	>1,024	64	1	>128	64	0.25	125
525	512	512	1,024	512	256	>1,024	>1,024	128	32	16	>128
527	1,024	512	1,024	>1,024	64	>1,024	>1,024	>128	>128	128	0.125
593	>1,024	>1,024	>1,024	>1,024	>1,024	128	64	>128	128	2	0.5
615	>1,024	>1,024	>1,024	>1,024	>1,024	>1,024	16	>128	>128	1	32
617	>1,024	>1,024	>1,024	>1,024	>1,024	>1,024	32	>128	>128	1	32
619	>1,024	>1,024	>1,024	>1,024	>1,024	>1,024	32	>128	>128	1	32

*ABK, arbekacin; AMK, amikacin; TOB, tobramycin; ISP, isepamycin; GEN, gentamicin; SM, streptomycin; NEO, neomycin; CTX, cefotaxime; CAZ, ceftazidime; IPM, imipenem; CIP, ciprofloxacin.

†MICs of kanamycin and sisomycin are not listed because values are >1,024 for all strain numbers. ‡See Table 2 for bacterial species and PCR type of 16S rRNA methylase gene of each strain number.

## Conclusions

The overall isolation frequency of 16S rRNA methylase-gene-positive gram-negative bacilli was very low (0.03%) in Japanese medical facilities in 2004, with the highest rates seen in *P. aeruginosa* and *Acinetobacter* spp. at 0.08% and 0.12%, respectively. Twenty-six bacterial isolates carrying 1 of the four 16S rRNA methylase genes were recovered from 16 (9.5%) of 169 hospitals that participated in this nationwide investigation. Of the 169 hospitals, 162 hospitals had >200 beds, accounting for 5.9% of all Japanese hospitals of similar scale. This implies that 16S rRNA methylase–producing strains might have been present in >250 Japanese hospitals during the investigation period, which in turn suggests sparse but diffuse spread of 16S rRNA methylase producers in Japan. Since several *armA-* or *rmtB*-positive strains have also been isolated in European and Asian countries, and given the potential for further dissemination, nationwide identification and ongoing surveillance of these isolates should be considered by all countries.

According to PFGE typing, nosocomial transmission of 16S rRNA methylase–producing *P. aeruginosa* and *A. baumannii* was suspected in 3 hospitals (hospitals C, D, and S). The banding patterns of *rmtA*-harboring *P. aeruginosa* isolated in hospitals C, D, and F were diverse, which excluded the possibility of an epidemic *P. aeruginosa* strain harboring the *rmtA* gene. Despite the observation of 2 different PFGE profiles among the 4 *P. aeruginosa* strains isolated in hospital D, they might share the same plasmids carrying the *rmtA* gene. For further characterization of genetic relations among *rmtA*-harboring *P*. *aeruginosa* strains, comparative analyses of plasmids and mobile elements that carry the *rmtA* gene (*14*) should also be pursued.

Nosocomial infections caused by multidrug-resistant, gram-negative bacteria have become a serious problem in clinical facilities. *P. aeruginosa* and *Acinetobacter* spp. have been especially efficient at developing multidrug resistance against broad-spectrum β-lactams, fluoroquinolones, and aminoglycosides (*3*,*6*,*7*,*9*). The identification of *armA* and *rmtB* genes in Europe and East Asia in both human (*1*–*11*) and livestock (*15**;* EMBL/GenBank accession no. DQ345788) populations suggests that we must pay consistent attention to prevent further global proliferation. If 16S rRNA methylase–positive bacterial isolates disseminate widely and extensively, the high level of pan-aminoglycoside resistance will undoubtedly have an impact on illness, deaths, and costs of care in both clinical and livestock-breeding environments.
